# Combination of Low Fluctuation of Temperature with TiO_2_ Photocatalytic/Ozone for the Quality Maintenance of Postharvest Peach

**DOI:** 10.3390/foods9020234

**Published:** 2020-02-21

**Authors:** Xiaoyu Jia, Jiangkuo Li, Meijun Du, Zhiyong Zhao, Jianxin Song, Weiqiao Yang, Yanli Zheng, Lan Chen, Xihong Li

**Affiliations:** 1 State Key Laboratory of Food Nutrition and Safety, College of Food Science and Engineering, Tianjin University of Science and Technology, Tianjin 300457, Chinasjxtust@126.com (J.S.); zhengyanli3344@163.com (Y.Z.); chenlan890804@163.com (L.C.); 2 Tianjin Key Laboratory of Postharvest Physiology and Storage of Agricultural Products, National Engineering and Technology Research Center for Preservation of Agricultural Products, Tianjin 300384, China; lijkuo@sina.com; 3 Instiute of Agro-Products Processing Science and Technology, Xinjiang Academy of Agricultural and Reclamation Science, Shihezi 832000, China; 4 Academy of National Food and Strategic Reserves Administration, Beijing 100037, China; 5 Tianjin Gasin-DH Preservation Technologies Co., Ltd., Tianjin 300403, China

**Keywords:** peach, chilling injury, internal circulation system, low fluctuation of temperature, TiO_2_ photocatalytic, storage quality

## Abstract

Chilling injury, tissue browning, and fungal infection are the major problems of peach fruit during post-harvest storage. In this study, a precise temperature control cold storage with low-temperature fluctuation (LFT) and internal circulation flow system is designed. An ozone (O_3_) generator and a (titanium dioxide) TiO_2_ photocatalytic reactor were applied to cold storage to investigate the variation of LFT combined with ozone fumigation and a TiO_2_ photocatalytic reactor in the efficiency of delaying ripening and maintaining peach fruit quality. Results showed that the temperature fluctuation with the improved control system was only ±0.1 to ±0.2 °C compared with that of ±0.5 to ±1.0 °C in conventional cold storage. LFT significantly reduced the chilling injury of peach fruit during storage. Although LFT combined with fumigation of 200 mg m^−3^ ozone periodical treatment slightly damaged the peach fruit after 40 d of storage, its combination with the TiO_2_ photocatalytic system significantly improved the postharvest storage quality of the fruit. This treatment maintained higher titratable acidity (TA), total soluble solids (TSS), better firmness, color, microstructure, and lower decay rate, polyphenol oxidase (PPO) activities, total phenol accumulation, respiratory intensity, ethylene production, and malondialdehyde (MDA) content during 60 d of storage. All the results show that LFT combined with the TiO_2_ photocatalytic system might be a promising technology for quality preservation in peach fruit storage.

## 1. Introduction

Peach [*Prunus persica* (L.) Batsch] belongs to the Rosaceae family, with a long history of cultivation in China. The output quantity of peach is more than 8 million tons every year [1]. It is worth noting that peach presents strong respiration intensity and rapid softening after harvest, which leads to short shelf-life [2]. Up to now, various techniques such as normal atmosphere cold storage, hypobaric storage, gamma-irradiation, heat treatments, and controlled atmosphere cold storage have been applied in peach preservation [3,4,5]. Among these technologies, cold storage is the most commonly used, especially for large amounts of peaches. However, during low-temperature storage, chilling injuries, quick softening, browning, woolly texture, and high perishability are still the main problems [6].

Accurate control of temperature and humidity in a cold storage room is generally not simple [7]. Non-uniform airflow from air coolers, evaporator defrosting processing, and frequent door opening and closing during storage result in the fresh products being exposed to undesired high-temperature fluctuations [8]. At present, temperature fluctuations in commercial cold storage rooms are approximately ±0.5 to ±1.0 °C [9]. Peach, like cucumber and mango, is sensitive to temperature fluctuation and prone to chilling injury during storage [10]. Chilling injury is mainly caused by the conversion of membrane lipids from liquid crystal phase to rigid solid gel phase, which destroys the integrity of the cell membrane [11]. Temperature also affects respiration and postharvest rot rates of the product [12]. The application of a jacketed storage system provided a feasible scheme to overcome these disadvantages, in which refrigerated air is circulated through an air space surrounding the storage room rather than within the space itself [13]. Although the construction cost of jacketed storage is 15% higher than that of conventional cold storage, the use of jacketed systems can significantly improve product quality and storage life, which depends on better temperature control and higher relative humidity [14,15,16,17]. In addition, the jacketed system can significantly reduce energy consumption because of sensible cooling during the dormant storage period and less defrosting times [13].

Titanium dioxide (TiO_2_) and ozone have been evaluated as promising sanitizers for fresh fruit and vegetables [18,19]. As a wide band gap (3.2 eV) semiconductor under ultroviolet (UV) (320–400 nm) illumination, TiO_2_ generates energy-rich electron-hole pairs that can be transferred to the surface of TiO_2_ and promotes reactivity with the surface-absorbed molecules leading to the production of active radicals [20]. TiO_2_ efficiently promotes the photocatalytic oxidation of organic compounds and the oxidation of microorganism cell membranes [21]. In practical application, TiO_2_ is mainly used in food packaging and photocatalytic reactors [19,22]. TiO_2_ photocatalytic reactors had been applied to tomato fruit during storage to delay ripening time [23]. Because of its ability to reduce microorganisms and for the oxidation of ethylene, gaseous ozone had been successfully applied for the storage of various fruits such as apples, papayas, orange potatoes, pears, and strawberries [24,25]. However, there was no research into the effect of LFT combined with ozone and a TiO_2_ photocatalytic system on postharvest quality of peaches.

Although both ozone and TiO_2_ photocatalysis treatments have been studied in a variety of fruit and vegetables, little has been done in their combination with low-temperature fluctuation for peach storage. Here, the improved temperature control and the combination of this technique with TiO_2_ photocatalysis or ozone intermittent treatment were investigated in the cold storage of peaches, and important quality parameters such as TSS, (polyphenol oxidase) PPO activity, total phenol content, (malondialdehyde) MDA, and fruit microstructure were analyzed in cold-stored peaches. The main purpose of this study is to evaluate the optimal temperature and post-harvest quality control techniques for cold storage of peaches.

## 2. Materials and Methods

### 2.1. Plant Material

Peaches [Prunus persica (L.) Batsch cv. Jin Qiu hong] were obtained from an orchard in Beijing, China. Only fruit free from damage, diseases or infestations, and of approximately a uniform size (250 to 300 g) and maturity were selected and pre-cooled to 5 °C [26].

### 2.2. Specification of Cold Storage

**Figure 1:**
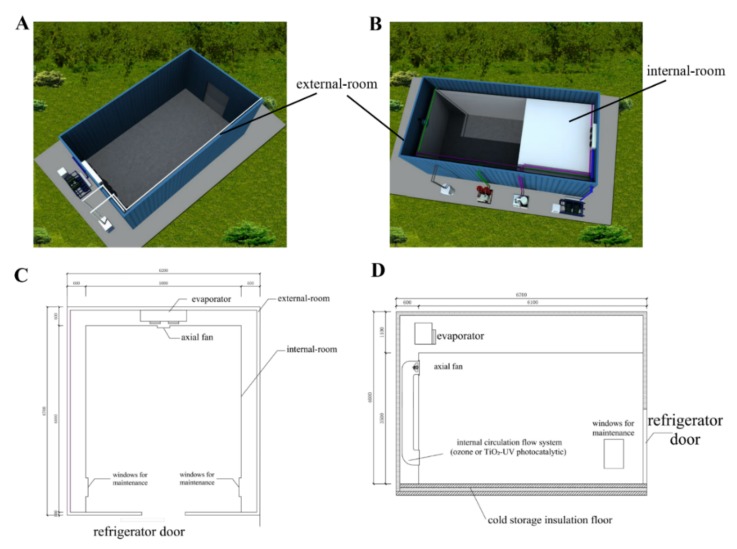
Comparison of conventional cold storage and LFT controlled cold storage. (**A**) Structure of conventional cold storage room. (**B**) Structure of LFT controlled cold storage room. (**C**) Floor plan of LFT cold storage room. (**D**) Horizontal profile of LFT controlled cold storage room.

The structure of conventional cold storage is shown in Figure 1A. The cold storage with improved precise temperature control is composed of both a separate external jacket and an internal storage room, refrigeration equipment, and an internal circulation flow system. The jacket-room (6700 × 6200 × 4600 mm) was a conventional cold store (Figure 1B), while the internal storage room (6000 × 5000 × 3500 mm) was made up of 1.5-mm thick aluminum plates. The fruit were placed in the internal room.

### 2.3. Gaseous Ozone and TiO_2_ Treatment

The ozone and TiO_2_ photocatalytic reactor were separately installed in the internal circulation system (Figure 1C,D). Ozone (90%) was produced by an FL-803Y ozone generator (Shenzhen Feili Electrical Technology Co. Ltd., Shenzhen, China). The JSDHMK-1227 TiO_2_ photocatalytic reactor was provided by a local company (Tianjin Gasin-DH Preservation Technologies Co. Ltd., Tianjin, China). The particle specific surface area and equivalent particle size of TiO_2_ were 11.7 m^2^ g^−1^ and 93.7 nm, respectively, the wavelength of ultraviolet light was 365 nm. The TiO_2_ coated surface area was 592.8 cm^2^.

### 2.4. Storage Condition

Peach samples were randomly divided into 40 plastic baskets (10 kg per basket). Peaches of each treatment (10 baskets) were transferred to the corresponding cold rooms, which were set as 0 °C. All cold storage rooms were built by Tianjin Gasin-DH Preservation Technologies Limited Co. Ltd (Tianjin, China). The treatments were as follows: CK (control), stored in a conventional cold storage room; LFT, stored in a precise temperature control cold storage; LFT + O_3_, stored in a precise temperature control cold storage and fumigated with ozone concentration of 200 mg m^−3^ (selected from previous experiments) for 30 min every week; LFT + TiO_2_, stored in a precise temperature control cold storage and treated with a TiO_2_ photocatalytic system for 30 min every week. The relative humidity for all treatments was 90% ± 5%. For each treatment, the experimental measurements were taken every ten days.

### 2.5. Analysis of Cold Storage Temperature Fluctuation

A BTC-A16 temperature and humidity recording instrument (Tianjin Boyuanda Technology Co. Ltd., Tianjin, China) was used to measure the temperature fluctuation in all storage rooms. The uniformity of temperature distribution in the conventional and the improved cold storage was evaluated at 35 temperature measurement points at heights of 0.5 and 3.5 m.

### 2.6. Analysis of Physicochemical Properties

#### 2.6.1. Color and Firmness Analysis

Fruit surface color was determined on three different locations of each individual fruit using the HP-200 colorimeter (Shanghai Chinaspec Optoelectronics Technology Co. Ltd., Guangzhou. China) [27]. The L* (lightness), a* (reddish-greenish) and b* (yellowish-bluish) indexes of the International Commission on Illumination Lab color space (CIELAB) colorimetric system were used to evaluate the color change of the peach samples [28].

The firmness of flesh fruit was measured using a GY-4 digital penetrometer (Zhejiang Top Instrument Co. Ltd., Hangzhou, China) with the needle-like probe of 10 mm diameter [29]. The results were expressed as Newton (N). Ten replicates were measured for this analysis, in which two fruit were used for each replicate.

#### 2.6.2. Titratable Acidity (TA) and Total Soluble Solids (TSS) Analysis

TA was measured by acid-base titration and expressed as a malic acid content percentage [30]. The total soluble solid (TSS) content of a peach was measured by a PAL-1 refractometer (Atago Co. Ltd., Tokyo, Japan) [31]. Ten replicates were performed for this analysis, in which two fruit were used for each replicate.

#### 2.6.3. Respiratory Rate and Ethylene Production

A GXH-305 infrared gas analyzer (Junfang Science & Technology Institute of Physical and Chemical Research, Beijing, China) was used to measure the respiratory rate, according to the method of Yang et al. [32]. Peach samples were placed in gas-tight jars for 1 h at 0 °C. Ethylene production was measured according to the method of Huan et al. [33]. The head space gas (1 mL) gas was injected into an Agilent GC 7890 gas chromatograph (Agilent Technologies, Santa Clara, CA, USA) equipped with a flame ionization detector (FID). Fresh mass-based rates of respiration were measured as CO_2_ release (mg kg^−1^ h^−1^) and those of ethylene production were given as µL kg^−1^ h^−1^.

#### 2.6.4. Decay Rate Analysis

The decay rate was measured by a previous method with visual evaluation [34]. The growth of mold on peach was regarded as decay, and the analysis carried out 10 repetitions based on 25 peaches.

#### 2.6.5. Polyphenol Oxidase (PPO) Activity and Total Phenolics Content Analysis

PPO activity was measured according to the method of Wang et al. [28]. Briefly, 3.0 g tissue was homogenized in 10 mL of 0.05 mol L^−1^ sodium phosphate buffer (pH 7.8) and 0.5 g of polyvinylpyrrolidone (PVPP). Then the homogenate was centrifuged in a 5804-r refrigerated centrifuge (Eppendorf, International Trade Co., Ltd., Shanghai, China) at 5000× *g* for 10 min. The supernatant (0.5 mL) was mixed with 1.5 mL phosphate buffer (0.05 mol L^−1^, pH 7.8) and 1 mL catechol (0.1 mol L^−1^). The change of optical density (OD) of the reaction mixture was measured every 30 s for 3 min at 420 nm by a 756-PC UV–vis spectrophotometer (T6 New Century, Beijing Purkinje General Instrument Co., Ltd., Beijing, China). The result of PPO activity was expressed as U g^−1^ fresh weight, where U = 0.01 Δ420 nm per min.

The Folin–Ciocalteu procedure was used to measure the total phenolic content according to the method of Piccolella et al. [35] with modifications. Briefly, 2.0 g of peach samples were crushed and added with 5.0 mL of 60% ethanol solution (*v*/*v*), then centrifuged at 3000× *g* for 10 min. Next, 0.25 mL of the supernatant was obtained and added with 5.0 mL of distilled water, 0.25 mL of Folin–Phenol reagent and 0.8 mL of 20% (*w*/*w*) Na_2_CO_3_, respectively. Then the mixture was placed in dark place for 30 min and measured at 760 nm absorbance. The results were expressed as g kg^−1^ fresh weight.

#### 2.6.6. Malondialdehyde (MDA) Content Analysis

Malondialdehyde (MDA) content was measured by thiobarbituric acid-reactive substance [36]. Peach tissue (5.0 g) was homogenized with 10 mL of trichloroacetic acid (TCA, 100 g L^−1^) and then centrifuged at 16,000× *g* for 10 min. The reaction mixture was a blend of 2.0 mL supernatant and 2.0 mL of thiobarbituric acid (0.5%, TBA), was heated in boiling water and cooled before centrifuged at 1000× *g* for 15 min. Finally, the absorbance of the mixture was measured at 532, 600, and 450 nm. MDA content was expressed on a fresh weight basis as mmol kg^−1^.

#### 2.6.7. Scanning Electron Microscope (SEM) Analysis

Microstructure of peach fruit was analysed with a SEM, as previously described [34]. The peach samples (approximately 3.0 × 3.0 × 1.0 mm) were cut from fruit with a blade. The samples were freeze-dried in a FD-1A-50 lyophilizer for 12 h (Shanghai billon instrument Co. Ltd., Shanghai, China) and then coated with 25 nm thick gold using a Balzers Union SCD 040 Sputter Coater (Balzer, Wiesbaden, Germany) before SEM analysis. Representative areas were examined with a Hitachi S-2500 (Hitachi Ltd., Tokyo, Japan) scanning electron microscopy at an accelerating voltage of 20 kV.

### 2.7. Statistical Analysis

Experiments were performed in a completely randomized manner with 10 replicates. SPSS version 13.0 software (SPSS Inc., Chicago, IL, USA) was used for the one-way analysis of variance (ANOVA) at the level of *p* < 0.05. All data was repeated ten times and expressed as mean ± SD (standard deviation).

## 3. Results and Discussion

### 3.1. Temperature Fluctuation

**Figure 2:**
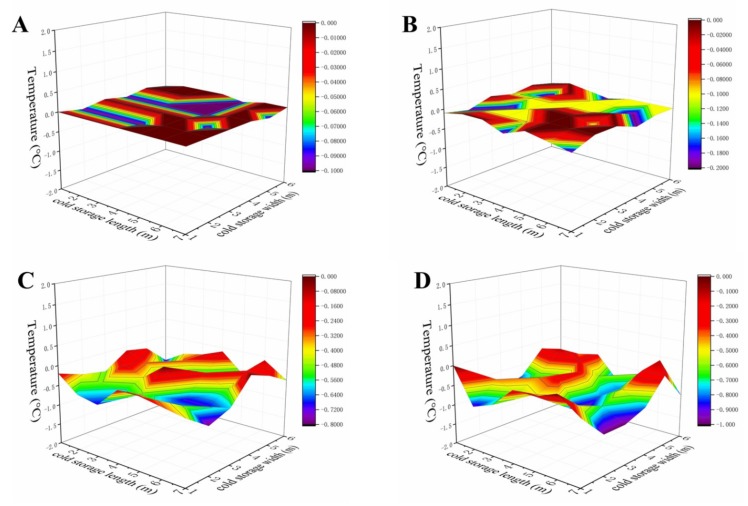
Temperature fluctuations at 0.5 (**A**,**C**) and 3.5 m (**B**,**D**) height in the improved (**A**,**C**) and the conventional (**B**,**D**) cold storage.

Random temperature fluctuations can cause the centre temperature of chilled produce to decrease temporarily below the threshold level beyond which cold injury may develop [37]. Moreover, temperature fluctuations may exacerbate moisture condensation, which lead to microbial growth and fruit rot [38]. In order to analyze the temperature fluctuation of the cold storage system, the temperature distribution of 0.5 and 3.5 m height planes in the conventional cold storage and the improved cold storage was measured. At a temperature setting of 0 °C, the temperature fluctuation in the jacketed storage room was small at only ±0.1 to ±0.2 °C (Figure 2A,B), if compared to ±0.5 to ±1.0 °C in the conventional cold store (Figure 2C,D). The latter was temperature-controlled by the refrigeration equipment (Figure 1A). Direct cooling was used and the products were exposed to the cold blown air [13]. In the improved cold storage system, the storage room was cooled by air flowing through an enclosed space or jacket surrounding the walls, floor, and ceiling rather than by direct circulation of air through the room (Figure 1B,C). By adding an inner structure, it avoided direct contact between the products and the evaporator, and maintained a low temperature fluctuation in the internal room. A similar jacket system was reported by Raghavan et al. [18] and used to store fresh carrots, which inhibited the loss of moisture and maintained postharvest quality after long-term storage [17].

### 3.2. Physicochemical Properties

#### 3.2.1. Color and Firmness

**Figure 3:**
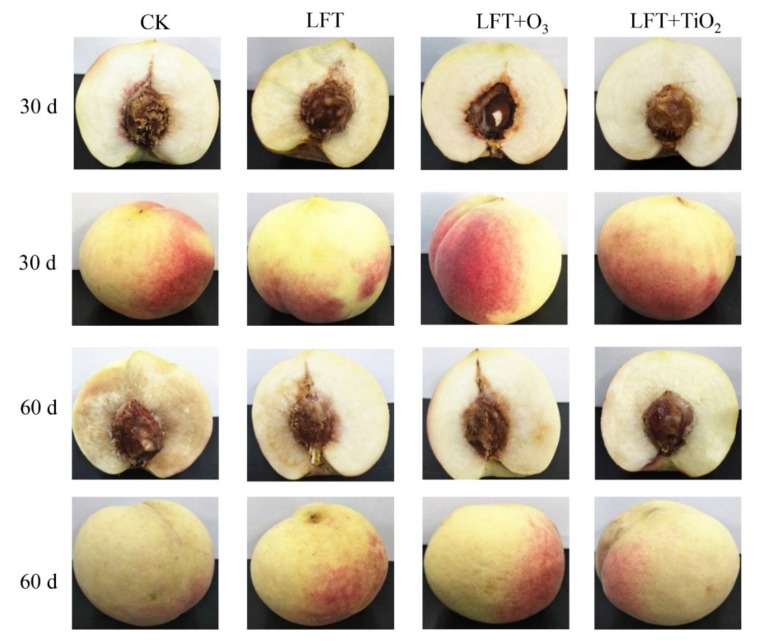
Effect of control (CK), LFT, LFT combined with ozone treatment (LFT + O_3_) and LFT combined with TiO_2_ photocatalysis (LFT + TiO_2_) treatments on the appearance and longitudinal section photos of peach fruit after storage of 30 and 60 d.

Figure 3 shows the appearance and section of peaches during storage for 30 and 60 d. For fruit from both cold store types, no significant differences in the color of the cut surfaces were found after 30 d of storage. In contrast, after 60 d, peaches of the CK group showed symptoms of severe chilling, i.e., browning, woolliness, and flesh translucency. The color of peach slices in LFT, LFT + O_3,_ and LFT + TiO_2_ groups were all better than of those of the CK group, which indicates that LTF significantly reduced the chilling damage of peach fruit during storage. On the skin of LFT + O_3_ peaches, symptoms of injury and pitted structures appeared after 60 d of storage. Similarly, high concentrations of gaseous ozone also caused damage to carrots [39]. Fruit of the LFT + TiO_2_ group showed the best appearance and bright flesh color (Figure 3). In agreement with the appearance observations, the L* value gradually decreased, whereas the b* value gradually increased throughout the storage of 60 d (Table 1). L* is the lightness and corresponds to a darkbright scale (0, black; 100, white). After storage for 20 d, L* value of the LFT, LFT + O_3_, and LFT + TiO_2_ peaches were higher than those of the CK group, indicating that LFT combined with either ozone fumigation or TiO_2_ photocatalysis could better maintain the L* value of peach fruit. Oddly, the L* value of peaches of the LFT + O_3_ group was lower than those of the LFT+TiO_2_ group after 40 d, which may be due to slight oxidation and damage of peach fruit caused by repeated ozone fumigation. A similar result was reported by Bridges et al. [40] where exposure to gaseous O_3_ at 1.71 mg g^−1^ for 5.0 h resulted in noticeable bleaching of carrot and tomato tissue. The increase of b* value indicates the deepening of yellow. In the experiment, the values of b* in the CK group were significantly higher than those in other treatment groups after 30 d. The LFT + TiO_2_ treatment showed the lowest b* value at 60 d, which expressed the lowest color change. Furthermore, the value of a* was not displayed due to no-significant differences between among different treatments.

The firmness of peach fruit in all groups continuously declining during storage; however, the firmness of fruits in LFT + O_3_ and LFT + TiO_2_ groups were significantly higher than that those of the CK and LFT groups after 40 d of storage (*p* < 0.05; Table 1). There was no significant difference between fruits in the LFT + O_3_ group and LFT + TiO_2_ group (*p* > 0.05). These results indicate that the combination of LFT and TiO_2_ photocatalysis or ozone treatment significantly impacts fruit firmness, which might be related to degraded ethylene during storage.

#### 3.2.2. Titratable Acidity (TA) and Total Soluble Solids (TSS)

TA values declined throughout the entire storage period for all treatments (Table 1). However, both LFT + O_3_ and LFT+TiO_2_ treatments delayed the loss of TA by 0.15% and 0.25%, respectively, during the 60 d of storage. Similar results were demonstrated by Ali et al. [41] and Li et al. [42] where papaya and strawberry fruit showed a decreasing tendency in TA value treated with ozone and TiO_2–_LDPE packaging, respectively.

TSS content is an important indicator of fruit maturity and intrinsic quality. There were no significant (*p* > 0.05) differences among all groups initially, but TSS content in CK group declined rapidly after 30 d. The TSS content in the LFT + TiO_2_ group increased from an initial value of 15.12% to 15.92% at 9 d of storage and then decreased to 13.20% at 60 d. However, due to the lack of a continuous supply of organic substances in the later period of storage, TSS can be consumed by respiration, resulting in the decline of TSS content [43]. At the end of storage (60 d), there was a significant difference among the TSS contents of the CK, LFT + TiO_2_, and LFT + O_3_ groups. These results were in accordance with the finding of Xing et al. [44] that the nano-TiO_2_ coating treatment could maintain mango quality by delaying the decline in TSS content.

#### 3.2.3. Respiratory Rate and Ethylene Production

**Figure 4:**
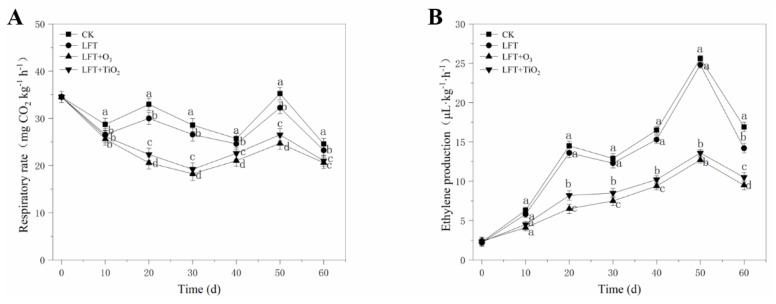
Effect of control (CK), LFT, LFT combined with ozone treatment (LFT + O_3_) and LFT combined with TiO_2_ photocatalytic (LFT + TiO_2_) treatments on the respiratory rate and ethylene production of peaches during storage at 0 °C for 60 d. Values are expressed as means ± SD (*n* = 10). Different letters (a–d) indicate significant differences among treatments for each sampling time at *p* < 0.05. (**A**) Respiratory rate. (**B**) Ethylene production.

As shown in Figure 4A, the CK and LFT treatment groups reached their first respiratory peak on the 20th day with 32.96 and 30 mg CO_2_ kg^−1^ h^−1^, respectively, while the LFT + O_3_ and LFT + TiO_2_ groups had only one respiratory peak on the 50th day of 24.68 and 26.52 mg CO_2_ kg^−1^ h^−1^. The CK and LFT treatment groups reached their maximum value of 35.25 and 32.25 mg CO_2_ kg^−1^ h^−1^, respectively, on the 50th day. In addition, the respiratory rate of LFT + O_3_ treated peaches was significantly lower than the other three groups from days 20 to 50 (*p* < 0.05). Ozone mainly inhibits the respiration by inhibiting oxidative phosphorylation of mitochondria of fruit cells and the normal electron-transport respiratory chain [45]. However, although the treatments of LFT + O_3_ and LFT + TiO_2_ were lower than the CK and LFT groups, there was no significant difference between them (*p* > 0.05). As a typical climacteric fruit, inhibiting or delaying the emergence of respiratory peak is the key measure to maintaining the storage quality of peach fruit [46]. It indicated that the combination of LFT and a TiO_2_ photocatalysis reactor or ozone could significantly inhibit the peach’s breathing. Similar results were reported by Han et al. [34] for ozone treatment on black mulberry, and Tao et al. [47] for a chitosan/nano-TiO_2_ composite film treatment on pears.

The release of ethylene could accelerate the ripening and senescence of peaches during storage [48]. As shown in Figure 4B, the maximum ethylene release of the LFT + O_3_ and LFT + TiO_2_ groups was 12.7 and 13.6 µL kg^−1^ h^−1^, respectively, which were significantly lower than of those of the CK and LFT groups at 40 d storage (*p* < 0.05). The difference in ethylene production between the CK group and the LFT + O_3_ group fruit was highly significant, and CK group fruit provided more than twice the ethylene production than that of LFT + O_3_ group fruit. Hawkins et al. [49] demonstrated that ozone and its anions have certain effects on endogenous ethylene degradation. In addition, our results show that LFT combined with ozone and TiO_2_ photocatalysis have a good synergistic effect in degrading ethylene during peach storage. Similar results have been reported in a previous study where the presence of TiO_2_ and UV-A light can remove ethylene gas from the storage atmosphere [23]. However, although the ethylene production of LFT treatment was also lower than that of the CK group, the differences were not significant (*p* > 0.05), which indicates that LFT has little effect on regulating the release of ethylene.

#### 3.2.4. PPO Activity and Total Phenolics Content

**Figure 5:**
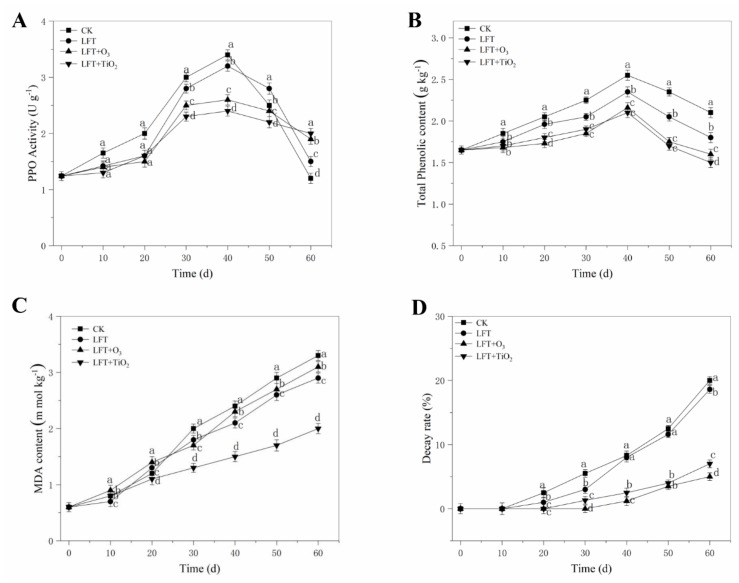
Effect of control (CK), LFT, LFT combined with ozone treatment (LFT + O_3_) and LFT combined with TiO_2_ photocatalytic (LFT + TiO_2_) treatments on the PPO activity, total phenolics content, decay rate, and MDA content of peaches fruit during storage at 0 °C for 60 d. Values are expressed as means ± SD (*n* = 10). Different letters (a–d) indicate significant differences among treatments for each sampling time at *p* < 0.05. (**A**) PPO activity. (**B**) Total phenolics content. (**C**) Malondialdehyde content. (**D**) Decay rate.

PPO has long been considered to be a major factor leading to fruit discoloration after harvest. As shown in Figure 5A, there was no significant difference in PPO activities among all treatments during the first 20 d of storage. However, PPO activity declined rapidly in both LFT treated and CK group fruit starting at 40 d, which might be because the fruit became senescent and over-ripe. Greater efficacies in inhibiting the PPO activities were found in both LFT + O_3_ and LFT + TiO_2_ treated fruit than that of CK group fruit after 20 d. Furthermore, at the 60th day of storage, reduction of PPO activity can be achieved by the LFT + TiO_2_ treatment, compared with the other treatments.

The total phenol content progressively increased for the first 40 d and then decreased during storage in all the treatments of peach fruit, and the fruit treated with LFT + TiO_2_ possessed the lowest total phenol content and the slowest increase rate (Figure 5B). This may be related to the TiO_2_ photocatalysis. A similar result was reported by Li et al. [42] that nano-TiO_2_–low-density polyethylene packaging could inhibit the biosynthesis of phenolics. As the substrate of enzymatic browning, the total phenol content exhibited positive correlated responses in the degree of browning [50]. Reducing the content of total phenol and PPO activity is one of the main modes of fruit resistance to browning [51]. Here, the LFT+O_3_ and LFT + TiO_2_ treatments can effectively reduce the formation of total phenols and inhibit the activity of PPO. In our study, higher PPO activity and total phenol content were found in LFT + O_3_ group fruit after 40 d of storage. It might be related to the physiological damage of the peach fruit being excessively exposed to ozone. The report of Ong et al. [52] showed that the balance between oxidative and reductive processes might be destroyed due to repeated ozone treatments, which then promotes the oxidation of phenolics, resulting in browning. Overall, LFT + TiO_2_ treatment could effectively reduce the PPO activity and the corresponding total phenolic content in peaches.

#### 3.2.5. Malondialdehyde (MDA) Content

As shown in Figure 5C, MDA content increased substantially in all treatments. However, the MDA content of peach fruit in the LFT + TiO_2_ group was remarkably restricted: only 60.6% of the decay rate of the CK group fruit at the end of storage time. LFT treatment reduced and delayed the accumulation of MDA, and MDA content in LFT treated fruit was 2.9 mmol kg^−1^ on day 60, showing about 12% less than that of the CK fruit. The cell membrane changes from a gel phase to a liquid crystal phase at large temperature fluctuations, which increases the risk of semi-permeable membrane loss [53]. However, higher MDA content of fruit was observed in the LFT+O_3_ treatment after 40 d, which may be due to the lack of free radical scavenging ability of ozone-treated fruits at low temperature [54]. In addition, the interaction of phenolic compounds with PPO is enhanced following damage of membrane integrity, which leads to tissue deterioration or senescence of the fruit [55]. In this study, it was observed that the LFT + TiO_2_ treatment reduced the MDA content. Our result is in agreement with a previous study, which reported that nano-TiO_2_ films can decrease the accumulation of MDA content of Ginkgo biloba seeds [56].

#### 3.2.6. Decay Rate

As shown in Figure 5D, fruits of the CK and LFT groups decayed during the first 20 d, and the degree of decay was significantly higher than of those of the LFT + O_3_ and LFT + TiO_2_ groups. However, the decay symptoms were observed after 30 d in fruits of LFT + TiO_2_ and LFT + O_3_ groups, and the decay rate of peach fruit was significantly reduced. The decay rates of LFT + TiO_2_ and LFT + O_3_ treatments were 65.2% and 75.3% lower than that of the control group at 60 d, respectively. Hoffmann et al. [57] reported that the superoxide anion radicals (·O_2_^-^) and hydroxyl radicals (·OH) produced by the TiO_2_ photocatalytic reactor under the irradiation of light with a specific wavelength have strong oxidative decomposition capability to kill bacteria by damaging the proteins in the cell membrane. Among all treatments, the LFT + O_3_ treatment employed in this study resulted in a significant effect on the decay rate of peach fruit, which might be related to the effective inhibition of ozone on microorganisms. Victorin [58] reported that ozone could destroy microorganisms by oxidizing cellular components such as sulfhydryl groups in amino acids and enzymes in cell membranes. Similarly, ozone could reduce the decay rate of blackberries after harvest [59].

#### 3.2.7. Scanning Electron Microscopy (SEM) Observation

**Figure 6:**
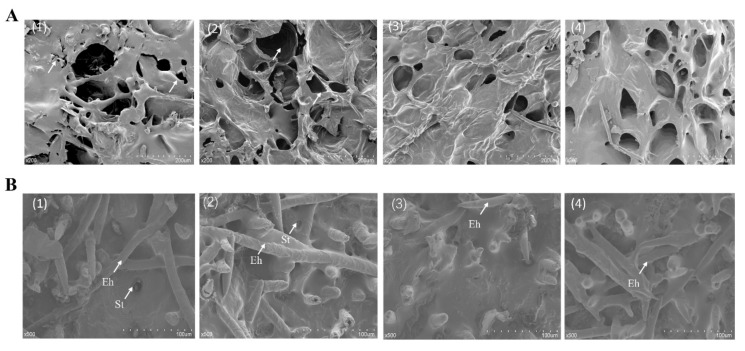
SEM images of different treatments on the flesh structure (**A**) and peel (**B**) of peach fruit on the 60th day. (**1**) CK; (**2**) LFT; (**3**) LFT + O_3_; (**4**) LFT + TiO_2_. The stomata and epidermis of peach skin are represented by St and Eh, respectively.

In this study, the micromorphology of the peel and flesh structure of peaches at the end of storage was observed. As shown in Figure 6A, significant destruction, folding, and deformation of flesh tissue were observed in the fruit of both the CK and the LFT groups at the end of storage. In contrast, LFT combined with ozone or a TiO_2_ photocatalytic reactor maintained an integrated and uniform tissue structure of peach fruit. This may reveal the ability of ozone and TiO_2_ photocatalysis to maintain normal physiological metabolism of peach fruit and inhibit microbial reproduction. SEM images of a control fruit surface-section showed deformed stomata, indicating the loss of moisture control function in epidermal cells (Figure 6B1). Conversely, the stomata were regular on the fruit surface of the LFT + TiO_2_ group, the morphology of guard cells was complete, the closed status could effectively suppress water loss (Figure 6B4). However, severe hair loss and stomatal closure occurred in the LFT + O_3_ treatment group due to repeated ozone fumigation. Similar phenomenon was also reported by Han et al. [34] where the stomas of black mulberry peel was closed by ozone treatment. In conclusion, the LFT + TiO_2_ photocatalytic treatment significantly suppressed the degradation of flesh and epidermal tissue of peach fruit, allowing the maintenance of their morphological features.

## 4. Conclusions

In the present study, low-temperature fluctuations combined with either ozone fumigation or a TiO_2_ photocatalysis reactor could effectively reduce the decay and respiration rates, and degrade ethylene during refrigerated storage. However, slight oxidation and damage of peach fruit were found in the LFT + O_3_ treatment during the later stage of storage. In addition, the LFT + TiO_2_ treatment was superior to LFT + O_3_ in maintaining fruit color and microstructure, inhibiting the enzyme activity of PPO, preventing the substrate generation of total phenol, and extending the shelf-life of peach fruit. In summary, the LFT + TiO_2_ treatment provided a more appropriate air composition for peach storage, which was conducive to prolonging the postharvest life and ensuring the quality of fruit during storage.

## Figures and Tables

**Table 1:** Changes of physicochemical properties of different treatments after 60 d storage.

Physicochemical Properties	Storage Period (d)	Treatments			
		CK	LFT	LFT + O_3_	LFT + TiO_2_
Color (L* value)	0	78.75 ± 0.12 ^a^	78.75 ± 0.09 ^a^	78.82 ± 0.08 ^a^	78.85 ± 0.05 ^a^
	10	76.47 ± 0.10 ^a^	76.49 ± 0.11 ^a^	76.53 ± 0.07 ^a^	76.81 ± 0.06 ^a^
	20	73.44 ± 0.13 ^c^	74.81 ± 0.10 ^b^	76.35 ± 0.05 ^a^	76.23 ± 0.06 ^a^
	30	72.56 ± 0.11 ^c^	74.20 ± 0.12 ^b^	75.60 ± 0.04 ^a^	74.86 ± 0.05 ^ab^
	40	68.54 ± 0.09 ^c^	72.35 ± 0.10 ^b^	73.25 ± 0.13 ^b^	74.56 ± 0.12 ^a^
	50	65.52 ± 0.13 ^d^	69.01 ± 0.09 ^c^	71.52 ± 0.08 ^b^	73.67 ± 0.12 ^a^
	60	59.95 ± 0.10 ^d^	63.79 ± 0.14 ^c^	70.46 ± 0.10 ^b^	72.43 ± 0.09 ^a^
Color (b* value)	0	5.25 ± 0.02 ^a^	5.16 ± 0.01 ^a^	5.21 ± 0.01 ^a^	5.23 ± 0.02 ^a^
	10	5.66 ± 0.01 ^a^	5.56 ± 0.02 ^a^	5.53 ± 0.03 ^a^	5.51 ± 0.04 ^a^
	20	6.01 ± 0.04 ^a^	5.92 ± 0.05 ^a^	5.96 ± 0.03 ^a^	5.94 ± 0.03 ^a^
	30	6.32 ± 0.05 ^a^	6.22 ± 0.02 ^a^	6.28 ± 0.03 ^a^	6.19 ± 0.02 ^a^
	40	7.82 ± 0.01 ^a^	6.86 ± 0.01 ^b^	6.57 ± 0.03 ^b^	6.35 ± 0.03 ^c^
	50	9.02 ± 0.04 ^a^	8.52 ± 0.01 ^b^	8.01 ± 0.02 ^c^	6.52 ± 0.03 ^d^
	60	10.53 ± 0.02 ^a^	9.54 ± 0.04 ^b^	8.85 ± 0.03 ^c^	6.68 ± 0.05 ^d^
Fruit Firmness (N)	0	58.72 ± 0.02 ^a^	58.62 ± 0.03 ^a^	58.71 ± 0.01 ^a^	58.63 ± 0.04 ^a^
	10	52.51 ± 0.03 ^a^	52.62 ± 0.03 ^a^	52.54 ± 0.04 ^a^	52.53 ± 0.03 ^a^
	20	46.03 ± 0.04 ^a^	47.41 ± 0.03 ^a^	48.32 ± 0.06 ^a^	47.44 ± 0.03 ^a^
	30	40.72 ± 0.03 ^b^	42.01 ± 0.04 ^b^	45.23 ± 0.05 ^a^	46.22 ± 0.06 ^a^
	40	36.31 ± 0.05 ^b^	38.81 ± 0.06 ^b^	43.12 ± 0.03 ^a^	44.13 ± 0.02 ^a^
	50	30.92 ± 0.04 ^c^	31.43 ± 0.03 ^c^	37.92 ± 0.02 ^b^	42.01 ± 0.03 ^a^
	60	25.62 ± 0.06 ^d^	29.31 ± 0.02 ^c^	35.91 ± 0.05 ^b^	41.82 ± 0.04 ^a^
Titratable Acidity (%)	0	0.42 ± 0.01 ^a^	0.42 ± 0.02 ^a^	0.42 ± 0.01 ^a^	0.42 ± 0.01 ^a^
	10	0.36 ± 0.02 ^a^	0.37 ± 0.00 ^a^	0.38 ± 0.01 ^a^	0.36 ± 0.01 ^a^
	20	0.28 ± 0.00 ^a^	0.29 ± 0.02 ^a^	0.36 ± 0.03 ^a^	0.34 ± 0.01 ^a^
	30	0.18 ± 0.01 ^d^	0.26 ± 0.02 ^b^	0.25 ± 0.03 ^c^	0.27 ± 0.02 ^a^
	40	0.12 ± 0.02 ^d^	0.20 ± 0.02 ^c^	0.22 ± 0.03 ^b^	0.21 ± 0.01 ^a^
	50	0.09 ± 0.01 ^d^	0.15 ± 0.02 ^c^	0.18 ± 0.00 ^b^	0.16 ± 0.01 ^a^
	60	0.06 ± 0.00 ^d^	0.12 ± 0.01 ^c^	0.15 ± 0.02 ^b^	0.14 ± 0.03 ^a^
Total Soluble Solids (%)	0	15.12 ±0.16 ^a^	15.12 ± 0.12 ^a^	15.12 ± 0.20 ^a^	15.12 ± 0.11 ^a^
	10	15.53 ± 0.14 ^a^	15.41 ± 0.12 ^a^	15.62 ± 0.18 ^a^	15.85 ± 0.19 ^a^
	20	15.83 ± 0.15 ^a^	15.65 ± 0.16 ^a^	15.85 ± 0.12 ^a^	15.92 ± 0.11 ^a^
	30	12.23 ± 0.10 ^b^	13.97 ± 0.11 ^a^	14.47 ± 0.14 ^a^	14.32 ± 0.10 ^a^
	40	11.36 ± 0.15 ^c^	13.05 ± 0.22 ^b^	14.32 ± 0.10 ^ab^	13.85 ± 0.08 ^a^
	50	10.85 ± 0.12 ^c^	12.51 ± 0.11 ^b^	13.22 ± 0.10 ^ab^	13.55 ± 0.21 ^a^
	60	9.81 ± 0.19 ^c^	11.32 ± 0.22 ^b^	12.23 ± 0.08 ^b^	13.20 ± 0.10 ^a^

* Means within each row with the different letters indicate significant difference (*p* < 0.05) between treatments.
